# Imaging Glycosylation In Vivo by Metabolic Labeling and Magnetic Resonance Imaging

**DOI:** 10.1002/ange.201509858

**Published:** 2015-12-03

**Authors:** André A. Neves, Yéléna A. Wainman, Alan Wright, Mikko I. Kettunen, Tiago B. Rodrigues, Sarah McGuire, De‐En Hu, Flaviu Bulat, Simonetta Geninatti Crich, Henning Stöckmann, Finian J. Leeper, Kevin M. Brindle

**Affiliations:** ^1^ Cancer Research UK Cambridge InstituteLi Ka Shing CentreCambridgeCB2 0REUK; ^2^ Department of ChemistryUniversity of CambridgeCambridgeCB2 1EWUK; ^3^ Department of Molecular Biotechnology and Health ScienceMolecular Imaging CenterVia Nizza 5210126TurinItaly; ^4^ A. I. Virtanen Institute for Molecular SciencesUniversity of Eastern FinlandNeulaniementie 270211KuopioFinland

**Keywords:** Bioorthogonale Chemie, Gadolinium, Glykane, Krebs, Magnetresonanztomographie

## Abstract

Glycosylation is a ubiquitous post‐translational modification, present in over 50 % of the proteins in the human genome,^1^ with important roles in cell–cell communication and migration. Interest in glycome profiling has increased with the realization that glycans can be used as biomarkers of many diseases,^2^ including cancer.^3^ We report here the first tomographic imaging of glycosylated tissues in live mice by using metabolic labeling and a gadolinium‐based bioorthogonal MRI probe. Significant *N*‐azidoacetylgalactosamine dependent *T*_1_ contrast was observed in vivo two hours after probe administration. Tumor, kidney, and liver showed significant contrast, and several other tissues, including the pancreas, spleen, heart, and intestines, showed a very high contrast (>10‐fold). This approach has the potential to enable the rapid and non‐invasive magnetic resonance imaging of glycosylated tissues in vivo in preclinical models of disease.

## Graphical abstract

**Glykane** wurden in lebenden Mäusen nach metabolischer Markierung mit einem Azidozucker und Zugabe eines an ein Gadolinium‐Kontrastmittel gebundenen gespannten Cyclooctins durch Magnetresonanztomographie dargestellt. Ein deutlicher Azidozucker‐abhängiger Kontrast wurde zwei Stunden nach Gabe des Kontrastmittels in verschiedenen Geweben beobachtet.

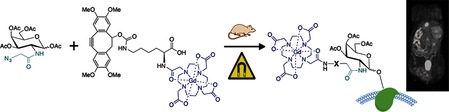

Various probes have been reported for imaging glycosylation in vivo, including antibodies,^4^ peptides,^5^ boronic acid derivatives,^6^ and lectins.^7^ Most approaches give a static view of cell‐surface glycosylation. We, and others, have used metabolic glycan labeling^8^ in combination with bioorthogonal reactions^9^ to image the dynamics of glycan biosynthesis in vivo. We demonstrated non‐invasive imaging of tumor glycosylation in live mice^10^ by metabolic labeling of tumor glycans with azido sugars followed by Staudinger ligation with a biotinylated phosphine and subsequent imaging using fluorescent or radionuclide‐labeled avidins. More recently, we described “double‐click” reagents,^11^ where azido‐modified cell‐surface glycoproteins were detected using a bivalent double‐click bioorthogonal probe. The latter consisted of a strained tetramethoxydibenzocyclooctyne (TMDIBO),^12^ which reacted specifically with azido sugar labeled glycans,^9^ and a *trans*‐cyclooctene (TCO), which reacted very rapidly with a fluorescently labeled tetrazine for fluorescence imaging (FLI). FLI gives high sensitivity and throughput;^13^ however, a limitation is light absorption and scattering, which prevents deep imaging in opaque organisms such as mice.

Recently, cells metabolically labeled with *N*‐azidoacetylmannosamine were imaged by magnetic resonance imaging (MRI) and a xenon (^129^Xe) biosensor. The azido group in cell‐surface sialic acid residues was detected using a bifunctional reagent incorporating bicyclo[6.1.0]nonyne, which reacted with the azido group, and a cryptophane, which bound hyperpolarized ^129^Xe. Bound xenon was detected by magnetization transfer measurements between free and bound xenon (hyper‐CEST).^14^ Although hyperpolarized ^129^Xe is very sensitive to MR detection, the change in signal intensity was relatively small (ca. 30–50 %), and only demonstrated for encapsulated cells in a bioreactor. A CEST‐based label‐free method for imaging underglycosylated mucin‐1 expression in vivo has also been described recently.^15^

Our aim was to develop a probe for the tomographic, non‐invasive MR imaging of metabolically labeled glycans in mice. A previously reported MRI probe, consisting of a phosphine conjugated to a gadolinium chelate,^11^ gave no detectable azido sugar dependent contrast in vivo owing to high levels of non‐specific binding, which we attributed to its hydrophobicity. We describe here TMDIBO–Lys–Gd (**2**; Figure 1), a novel water‐soluble probe that combines a strained cyclooctyne TMDIBO linked, via a hydrophilic lysine linker, to a gadolinium DOTA chelate, a clinically approved MRI contrast agent.^16^ This probe was used to image metabolically labeled cell‐surface glycans on tumor cells in vitro and in vivo. The probe also showed significant labeling of other mouse tissues, including the pancreas, spleen, kidney, liver, and gut.

**Figure 1:**
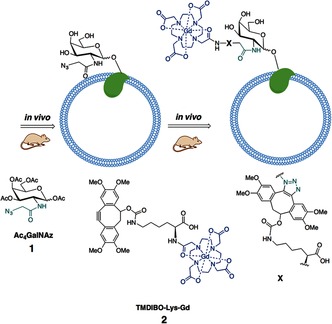
Labeling of cell‐surface glycans with an MRI‐detectable probe. Mice were injected daily with peracetylated *N*‐azidoacetylgalactosamine (Ac_4_GalNAz, **1**). Azido‐labeled cell‐surface glycoproteins were then detected in vivo by subsequent reaction with a bioorthogonal MRI contrast agent, TMDIBO–Lys–Gd (**2**).

The *T*_1_ relaxivity of **2** (Figure 1) in buffer at 7 T was 6.3±0.1 mm ^−1^s^−1^, similar to published values for Gd DOTA complexes.^16^ The reactivity of **2** with cell‐surface azido sugar labeled glycans was determined by culturing Lewis lung (LL2) adenocarcinoma cells with *N*‐azidoacetylgalactosamine (Ac_4_GalNAz, **1**; Figure 1) for 24 h, and then incubating them with **2** for 45 min, after which the cells were washed. The *R*_1_ (1/*T*_1_) relaxation rates were measured in pelleted cells that had been incubated with (+/+/−) or without (+/−/−) **1** and/or **2** (+/+/+, +/−/+; Figure 2). There was a significant increase (*P*<0.005) in *R*_1_ in azido sugar (**1**) treated LL2 cells that had been incubated with **2** (+/+/+, 1.38±0.10 s^−1^), when compared with cells not cultured with the azido sugar (+/−/+, 0.79±0.02 s^−1^) or with cells not incubated with either **1** or **2** (+/−/−, 0.64±0.03 s^−1^, *P*<0.001). The small increase in *R*_1_ (by a factor of 1.23±0.01) between cells that had not been treated with either **1** or **2** (+/−/−) and cells incubated with **2** alone (+/−/+) showed that there were only low levels of non‐specific binding of **2**. This was considerably less than observed previously with a fluorescently labeled version of TMDIBO, where this ratio was 3.3±0.1.^11^

**Figure 2:**
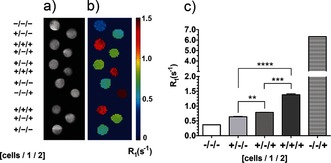
MR imaging of cell‐surface glycosylation in vitro. a) *T*_2_‐weighted images and b) corresponding maps of the *R*_1_ relaxation rates (1/*T*_1_). The images were obtained from cell pellets of untreated cells (+/−/−), cells treated with solvent vehicle and **2** (1.0 mm, for 45 min at 37 °C; +/−/+), or cells incubated with **1** (50 μm, for 24 h at 37 °C) and **2** (1.0 mm, for 45 min at 37 °C; +/+/+). The *R*_1_ rates were also measured in the buffer in which the cells had been suspended (−/−/−) and in a buffer to which **2** had been added (1.0 mm; −/−/+). Data represent the mean±standard error of the mean (SEM) (*n*=3). **P<0.01, ***P<0.005, ****P<0.001. Two‐tailed unpaired T‐test with Mann–Whitney correction.

Mice with flank tumors, obtained by subcutaneous injection of LL2 cells, were injected daily, for three days, with Ac_4_GalNAz (**1**; 300 mg kg^−1^, i.p.) or with solvent vehicle, and then injected with **2** (0.25 mmol kg^−1^, i.v.; gadolinium‐based contrast media are used clinically at 0.1–0.3 mmol kg^−1^) on day 4.^17^ The metabolic labeling of glycans with **1** and subsequent bioorthogonal detection with **2** was confirmed by ICP‐MS measurements of the gadolinium content in excised tissues (Figure 3) obtained after the imaging experiments (Figures 4 and 5), and 24 h after the injection of **2**. Most tissues showed *N*‐Ac_4_GalNAz dependent labeling. The gadolinium content was highest in the kidney (29.9±5.8 nmol Gd per gram of tissue). However, about one third of this was due to the non‐specific retention of **2** and thus not *N*‐Ac_4_GalNAz dependent (10.9±2.2 nmol Gd per gram of tissue), and metabolic labeling was relatively modest (2.7±0.5‐fold increase in gadolinium content in animals injected with **1** and **2** (+/+) compared with those injected with **2** alone (−/+)). The high background signal in the kidney is likely due to this organ being the preferred clearance route for molecules <1 kDa. The liver also showed relatively high levels of non‐specific retention of **2** (4.5±0.8 nmol Gd per gram of tissue) and similar levels of *N*‐Ac_4_GalNAz dependent labeling as the kidney (2.3±0.3‐fold). The levels of kidney and liver labeling are in agreement with our previous work on FLI.^11^ Other tissues showed very low levels of non‐specific background retention of **2** and consequently high levels of *N*‐Ac_4_GalNAz dependent labeling. The gadolinium concentration ratios for tissues from animals injected with *N*‐Ac_4_GalNAz (+/+) relative to those injected with the solvent vehicle (−/+) were 57±8 for the heart, 39±4 for spleen, 14±2 for the pancreas and the small and large intestine, and 5±1 for the lungs. Tumors showed much higher levels of non‐specific retention of **2**, and therefore, the gadolinium concentration ratio was much lower (3.3±1.1). Glycan‐labeling methods based on the delivery of labeled sugars using targeted liposomes^18^ could be used to improve tumor selectivity.

**Figure 3:**
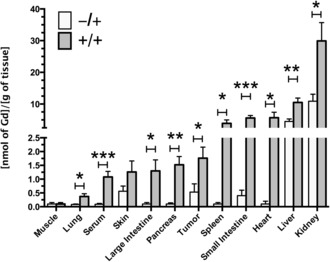
Gadolinium concentrations in metabolically labeled mouse tissues. The Gd content of excised tissues was determined by inductively coupled plasma mass spectrometry (ICP‐MS). Tissues were obtained from control mice (−/+) and Ac_4_GalNAz treated mice (+/+) after the imaging experiments and 24 h after injection of **2** (Figure 1). The Gd content was normalized to the wet tissue weight. Data represent the mean±SEM (*n*=5). *P<0.05, **P<0.01, ***P<0.005. Two‐tailed unpaired T‐test with Mann–Whitney correction.

Serum showed significant metabolic labeling (the serum gadolinium concentration ratio for animals injected with *N*‐Ac_4_GalNAz and those injected with the solvent vehicle was 12±1, *P*<0.005; Figure 3). Metabolic labeling of mouse serum^19^ is thought to result from the incorporation of azido sugars into the major glycosylated proteins present.^20^ We estimated the contribution of labeled serum glycoproteins to labeling of the small intestine, spleen, kidney, and liver from the serum contents of these tissues, which have been estimated to be 5.0, 9.2, 19.1, and 20.2 % of the tissue volume, respectively.^21^ The contribution of labeled serum proteins was estimated to be only 1.0, 2.4, 1.0, and 3.4 % of the total tissue glycan labeling, respectively. Tumors have a much larger interstitial volume fraction (20–40 %),^22^ and their leaky neovasculature results in the retention of macromolecules.^23^ However, the contribution to tumor labeling, due to retention of labeled serum proteins, was estimated to be only 15–30% of the total.

*T*_2_‐weighted images and *T*_1_ relaxation rate (*R*_1_) maps (Figure 4) were acquired in vivo before and at 2 and 24 h post injection of **2**. The relaxation rates (*R*_1_) for water protons in the tumor, kidney, and liver (Figure 4) were significantly higher in animals injected with **1** and **2** (+/+) than for the controls injected with the vehicle and **2** (−/+), demonstrating non‐invasive MRI detection of *N*‐Ac_4_GalNAz dependent tissue labeling in vivo (Figure 4 b–k).

**Figure 4:**
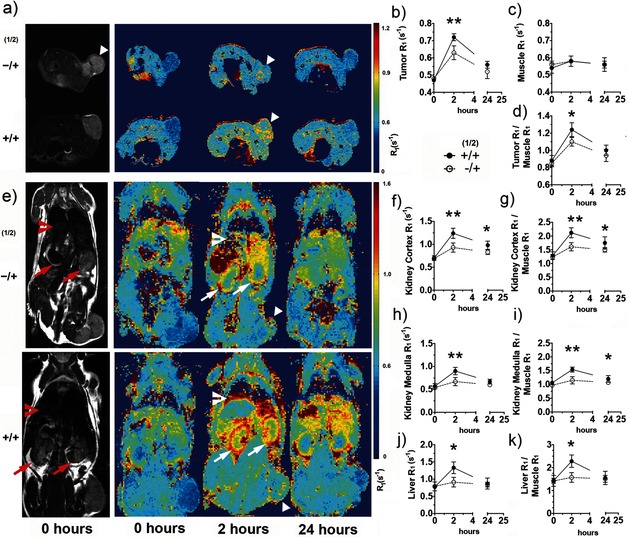
Imaging tissue glycosylation in vivo using MRI *T*_1_ maps. *T*_2_‐weighted (gray scale) images (a, e) and *T*_1_ maps (pseudo‐colored; a, e) of representative mice injected with the vehicle and **2** (−/+) or **1** and **2** (+/+), showing metabolic labeling of tumor (triangles), kidney (arrows), and liver (chevrons), 2 and 24 h after injection of **2**. a) Axial and e) coronal images are shown. The kinetics of *N*‐Ac_4_GalNAz dependent contrast formation were analyzed for regions of interest defined on the *T*_2_‐weighted images for the tumor (b), muscle (c), kidney cortex (f), medulla (h), and liver (j), and the corresponding data were normalized to the muscle data (d, g, i, k). Data in (b)–(d) and (f)–(k) represent mean±SEM (*n*=5). The relaxation rate *R*_1_ (1/*T*_1_) is expressed in s^−1^. *P<0.05, **P<0.01. Two‐tailed unpaired T‐test with Mann–Whitney correction.

The tumor contrast observed 2 h after probe injection was similar to that reported recently at 24 h after administration of a boronic acid based MRI contrast agent for detecting sialic acid in a murine model of melanoma.^6^ Other organs also showed appreciable labeling (Figure 4 e, +/+); however, analysis of the *T*_1_ maps was difficult owing to organ motion. A semi‐quantitative estimate of labeling based on coronal *T*_1_‐weighted images acquired in vivo (Figure 5 and Movie S1) 2 h post administration of **2** indicated significant azido sugar dependent labeling of tumor (*P*<0.05), kidney (*P*<0.05), gut (*P*<0.005), liver (*P*<0.05), and spleen (*P*<0.005).

**Figure 5:**
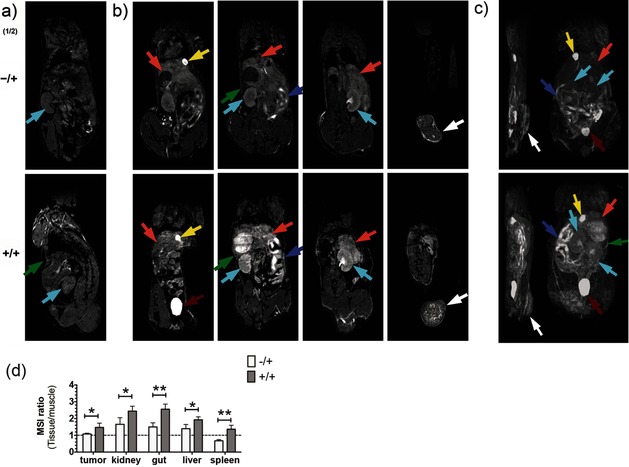
Imaging tissue glycosylation in vivo using *T*_1_‐weighted MRI. Coronal *T*_1_‐weighted images, before (a) and 2 h after (b) the injection of **2**. Maximum intensity projection of *T*_1_‐weighted signals (c) from representative mice injected with solvent vehicle and **2** (−/+) or **1** and **2** (+/+). Coronal *T*_1_‐weighted images (b, left to right) are displayed from the ventral towards the dorsal side. Maximum intensity projections (c) onto the sagittal (left) and coronal (right) planes. b) Metabolic labeling was observed in the tumor (white arrows), kidney (cyan), liver (orange), gut (purple), and spleen (green) 2 h after the injection of **2**. Bladder and gallbladder are indicated by red and yellow arrows, respectively. d) The *N*‐Ac_4_GalNAz dependent contrast was analyzed semi‐quantitatively, using regions of interest defined in the *T*_1_‐weighted images for the tumor, kidney, gut, liver, and spleen; the mean signal intensity (MSI) for these tissues was divided by the MSI of muscle. Data represent mean±SEM (*n*=4). *P<0.05, **P<0.005. Two‐tailed unpaired T‐test with Mann–Whitney correction.

The gadolinium concentrations in tumor, kidney, and liver were estimated using the *T*_1_ relaxivity of **2**, and the relaxation rates of these tissues measured in vivo (Figure 4). This gave gadolinium concentrations of 42±3, 80±6 and 93±12 μm, respectively, at 2 h post administration of **2** and 15±1, 38±3, and 17±1 μm at 24 h. The gadolinium concentration in the kidney measured ex vivo after 24 h was 30±6 μm (Figure 3), which is comparable with that estimated by the in vivo experiment. There was poorer agreement between the estimated in vivo concentrations in the tumor and liver at 24 h and those measured ex vivo, which were 1.8±0.4 and 11±1 μm, respectively (Figure 3). However, this can be explained by the fact that the estimated minimum MRI‐detectable tissue concentration of gadolinium is approximately 10 μm.^24^

In summary, the gadolinium‐labeled bioorthogonal probe described here can be used for the non‐invasive in vivo imaging of tissue glycosylation by magnetic resonance imaging. Most tissues showed only low levels of non‐specific retention of TMDIBO–Lys–Gd (**2**), and a significant *N*‐azidoacetylgalactosamine dependent contrast was observed within two hours of probe administration. As altered cell‐surface glycosylation is a hallmark of disease, particularly cancer, and MRI is a widely used imaging technique, this novel method may enable the rapid assessment of disease‐related changes in glycosylation in vivo.

## Supporting information

As a service to our authors and readers, this journal provides supporting information supplied by the authors. Such materials are peer reviewed and may be re‐organized for online delivery, but are not copy‐edited or typeset. Technical support issues arising from supporting information (other than missing files) should be addressed to the authors.

Supplementary

Supplementary

## References

[1] M. N. Christiansen , J. Chik , L. Lee , M. Anugraham , J. L. Abrahams , N. H. Packer , Proteomics 2014, 14, 525–546. 24339177 10.1002/pmic.201300387

[2] C. B. Lebrilla , H. J. An , Mol. Biosyst. 2009, 5, 17–20. 19081926 10.1039/b811781k

[3a] H. J. An , S. R. Kronewitter , M. L. de Leoz , C. B. Lebrilla , Curr. Opin. Chem. Biol. 2009, 13, 601; 19775929 10.1016/j.cbpa.2009.08.015 PMC2788081

[3b] D. W. Kufe , Nat. Rev. Cancer 2009, 9, 874. 19935676 10.1038/nrc2761 PMC2951677

[4] S. T. Laughlin , C. R. Bertozzi , Proc. Natl. Acad. Sci. USA 2009, 106, 12–17. 19104067 10.1073/pnas.0811481106 PMC2629201

[5] P. L. Hsiung et al., Nat. Med. 2008, 14, 454–458. 18345013 10.1038/nm1692 PMC3324975

[6] S. Geninatti Crich , D. Alberti , I. Szabo , S. Aime , K. Djanashvili , Angew. Chem. Int. Ed. 2013, 52, 1161–1164; 10.1002/anie.201207131 23225599

[7] E. L. Bird-Lieberman , A. A. Neves , P. Lao-Sirieix , M. O'Donovan , M. Novelli , L. B. Lovat , W. S. Eng , L. K. Mahal , K. M. Brindle , R. C. Fitzgerald , Nat. Med. 2012, 18, 315–321. 22245781 10.1038/nm.2616

[8] J. A. Prescher , D. H. Dube , C. R. Bertozzi , Nature 2004, 430, 873–877. 15318217 10.1038/nature02791

[9] C. P. Ramil , Q. Lin , Chem. Commun. 2013, 49, 11007–11022. 10.1039/c3cc44272a PMC3847904 24145483

[10a] A. A. Neves , H. Stöckmann , R. R. Harmston , H. J. Pryor , I. S. Alam , H. Ireland-Zecchini , D. Y. Lewis , S. K. Lyons , F. J. Leeper , K. M. Brindle , FASEB J. 2011, 25, 2528; 21493886 10.1096/fj.10-178590

[10b] R. Rossin , M. S. Robillard , Curr. Opin. Chem. Biol. 2014, 21, 161. 25159021 10.1016/j.cbpa.2014.07.023

[11] A. A. Neves , H. Stockmann , Y. A. Wainman , J. C. Kuo , S. Fawcett , F. J. Leeper , K. M. Brindle , Bioconjugate Chem. 2013, 24, 934–941. 10.1021/bc300621n PMC3687584 23642228

[12] H. Stöckmann , A. A. Neves , S. Stairs , H. Ireland-Zecchini , K. M. Brindle , F. J. Leeper , Chem. Sci. 2011, 2, 932–936. 22724056 10.1039/C0SC00631A PMC3378185

[13] S. Kumar , R. Richards-Kortum , Nanomedicine 2006, 1, 23–30. 17716206 10.2217/17435889.1.1.23

[14] C. Witte , V. Martos , H. M. Rose , S. Reinke , S. Klippel , L. Schröder , C. P. Hackenberger , Angew. Chem. Int. Ed. 2015, 54, 2806–2810; 10.1002/anie.201410573 25676513

[15] X. Song , R. D. Airan , D. R. Arifin , A. Bar-Shir , D. K. Kadayakkara , G. Liu , A. A. Gilad , P. C. van Zijl , M. T. McMahon , J. W. Bulte , Nat. Commun. 2015, 6, 6719. 25813863 10.1038/ncomms7719 PMC4380237

[16] P. Caravan , J. J. Ellison , T. J. McMurry , R. B. Lauffer , Chem. Rev. 1999, 99, 2293–2352. 11749483 10.1021/cr980440x

[17] M. F. Bellin , Eur. J. Radiol. 2006, 60, 314–323. 17005349 10.1016/j.ejrad.2006.06.021

[18a] R. Xie , S. Hong , L. Feng , J. Rong , X. Chen , J. Am. Chem. Soc. 2012, 134, 9914; 22646989 10.1021/ja303853y

[18b] R. Xie , L. Dong , R. Huang , S. Hong , R. Lei , X. Chen , Angew. Chem. Int. Ed. 2014, 53, 14082; 10.1002/anie.201408442 25330766

[19] P. V. Chang , J. A. Prescher , E. M. Sletten , J. M. Baskin , I. A. Miller , N. J. Agard , A. Lo , C. R. Bertozzi , Proc. Natl. Acad. Sci. USA 2010, 107, 1821–1826. 20080615 10.1073/pnas.0911116107 PMC2836626

[20] Y. Kita , Y. Miura , J. Furukawa , M. Nakano , Y. Shinohara , M. Ohno , A. Takimoto , S. Nishimura , Mol. Cell. Proteomics 2007, 6, 1437–1445. 17522412 10.1074/mcp.T600063-MCP200

[21] N. Kaliss , D. Pressman , Proc. Soc. Exp. Biol. Med. 1950, 75, 16–20. 14797719 10.3181/00379727-75-18083

[22] R. Weissleder , H. C. Cheng , E. Marecos , K. Kwong , A. J. Bogdanov , Eur. J. Cancer 1998, 34, 1448–1454. 9849430 10.1016/s0959-8049(98)00195-6

[23] H. Maeda , J. Wu , T. Sawa , Y. Matsumura , K. Hori , J. Controlled Release 2000, 65, 271–284. 10.1016/s0168-3659(99)00248-5 10699287

[24] S. Aime , C. Cabella , S. Colombatto , S. G. Crich , E. Gianolio , F. Maggioni , J. Magn. Reson. Imaging 2002, 16, 394–406. 12353255 10.1002/jmri.10180

